# Relationship between Geriatric Nutritional Risk Index with all-cause and CVD mortality in osteopenia and osteoporosis

**DOI:** 10.3389/fpubh.2024.1420832

**Published:** 2024-11-27

**Authors:** Tianting Guo, Haorong Feng, Lijiao Xiong, Jianwen Mo, Xiaoan Zhang, Junbin Xie, Hongkai Hu

**Affiliations:** ^1^Guangdong Provincial People's Hospital Ganzhou Hospital (Ganzhou Municipal Hospital), Ganzhou, Jiangxi, China; ^2^Department of Anesthesiology, South Taihu Hospital Affiliated to Huzhou College (Huzhou South Taihu Hospital), Huzhou, Zhejiang, China; ^3^The Second Clinical Medical College, Jinan University (Shenzhen People's Hospital), Shenzhen, China; ^4^The First Affiliated Hospital of Gannan Medical University, Ganzhou, Jiangxi, China

**Keywords:** osteopenia, osteoporosis, Geriatric Nutritional Risk Index, all-cause mortality, CVD mortality

## Abstract

**Purpose:**

This study investigated the relationship between Geriatric Nutritional Risk Index (GNRI), and all-cause, cardiovascular (CVD) mortality, in individuals with osteopenia and osteoporosis.

**Methods:**

Using NHANES data from 2005 to 2019, 6,824 participants diagnosed with osteopenia and osteoporosis were analyzed. Participants were categorized based on GNRI tertiles, and statistical methods like the fitted curves, Kaplan-Meier curves, cox regression, and subgroup analyses were employed.

**Results:**

Lower GNRI tertiles correlated with older age, male gender, and more comorbidities. Mortality rates differed significantly across GNRI tertiles over an average 7.9-year follow-up, with a notable inverse J-shaped association between GNRI and mortality. Adjusted HRs indicated a 1.6-2-fold increase in all-cause mortality for the lowest GNRI tertile, persisting across comprehensive adjustments. CVD mortality followed a similar trend.

**Conclusions:**

This study illuminates a robust correlation between GNRI levels and mortality risks in osteopenia and osteoporosis. Its consistent dose-response relationship across all-cause, and CVD mortality underscores its pivotal role as a prognostic factor.

## Introduction

Osteoporosis and osteopenia pose substantial health challenges globally, especially within aging populations (1). The prevalence of osteoporosis is escalating, affecting millions worldwide. In the United States alone, over 10 million individuals aged 50 and above suffer from osteoporosis, with an additional 34 million at risk of developing the condition (2, 3). The economic burden is substantial, amounting to ~$17.9 billion annually (2, 3). Recognizing the intricate interplay between nutritional status, osteoporosis, and mortality has become increasingly crucial. The Geriatric Nutritional Risk Index (GNRI), integrating serum albumin and body weight, stands out as a valuable tool for evaluating the nutritional status of older individuals (4, 5).

Established as an independent risk factor for osteoporosis in the older adult and postmenopausal women, GNRI demonstrates a negative and non-linear association with osteoporosis risk in these populations (4, 6). However, a research gap exists in exploring the connection between GNRI and mortality outcomes among individuals with osteoporosis. Given the rising global prevalence of osteoporotic conditions, decoding this relationship is paramount for tailored interventions and precise risk stratification. By leveraging NHANES data, we provide a nationally representative perspective on the convergence of nutrition, bone health, and mortality, with particular emphasis on CVD mortality. Our findings not only affirm established connections but also underscore the central role of GNRI in influencing critical endpoints such as all-cause mortality and CVD mortality.

## Materials and methods

### Data sources and preparation

The study utilized data from the National Health and Nutrition Examination Survey (NHANES), a nationally representative research initiative in the United States (7). NHANES enrolled non-institutionalized individuals who provided consent and underwent standardized questionnaires and physical examinations (7, 8). Data were collected from 2005 to 2019, with updated datasets released biennially and accessible at https://www.cdc.gov/nchs/nhanes/index.htm.

### Osteoporosis and osteopenia

In the assessment of osteoporosis and osteopenia, T-scores were computed using the formula (BMD_participant_ – mean BMD_reference_)/SD_reference_. The femoral neck T-score reference group, following World Health Organization guidelines, comprised non-Hispanic white females aged 20–29 from the NHANES III report, while the lumbar spine T-score reference group was derived from the CDC's Vital and Health Statistics (9–11). Osteopenia was defined by T-scores falling between −1.0 and −2.5, whereas osteoporosis was identified by T-scores below −2.5 (10–12).

### Study outcomes

This study focused on all-cause and cardiovascular (CVD) mortality endpoints. All-cause mortality included participant deaths from any cause between the baseline survey and December 31, 2019. Mortality data, obtained from the NHANES Public-use Linked Mortality Files (https://www.cdc.gov/nchs/data-linkage/mortality.htm), used precise International Classification of Diseases, Tenth Revision (ICD-10) codes (8). CVD mortality utilized specific ICD codes (054-068) (8), covering conditions like coronary artery disease, hypertensive heart disease, rheumatic heart diseases, ischemic heart diseases, pericardium disease, acute myocarditis, heart failure, and other heart diseases.

### GNRI calculation

The GNRI was determined using serum albumin (g/L), ideal body weight (kg), and actual body weight (kg). The formula employed was GNRI = (1.489 ^*^ serum albumin) + (41.7 ^*^ body weight/ideal weight) (4–6, 13, 14). The GNRI and Ideal weight was calculated with the formula described in the previous studies (4–6, 13, 14). These parameters were integral to the baseline examination during registration, ensuring methodological precision in assessing nutritional risk among the geriatric cohort.

### Covariates

Our investigation delved into a comprehensive array of factors. Demographic considerations comprised age, gender, and body mass index (BMI), while sociocultural facets were represented by race, ethnicity, education, and marital status. The intricate dynamics of race and ethnicity were captured by categorizing participants into five distinct groups: non-Hispanic White, Black, Mexican American, other Hispanic, or other (multiracial). Education levels, a key sociodemographic determinant, were stratified into four tiers: less than high school, high school graduate or equivalent, Some College or AA degree, and college graduate or above (8). Marital status added another layer of complexity, classified into Single, Married, Widowed, or Divorced or Separated. The profound impact of chronic diseases on our study was underscored through meticulous consideration of physician-diagnosed conditions, including coronary heart disease, diabetes, hypertension, chronic kidney disease, metabolic syndrome, stroke, chronic obstructive pulmonary disease (COPD), Parkinson's disease, and hyperlipidemia.

### Statistical analysis

Continuous variables were reported with 95% confidence intervals, while categorical variables were expressed as percentage frequencies. T-tests and χ2 tests facilitated comparative analyses, and the absence of imputation methodologies emphasized data completeness. Mortality risk assessment utilized Cox proportional hazards regression models, complemented by visual tools—curve fitting, Kaplan-Meier, and ROC curves. R software, the nhanesR package, and Free Statistics software version 1.9 supported these analyses. A two-sided *P*-value <0.05 was the threshold for statistical significance, aligning with rigorous standards. This streamlined approach ensures the reliability of our findings in this study.

## Results

### Demographics

The comprehensive exploration of baseline characteristics among 6,824 individuals with osteoporosis and osteopenia in NHANES 2005–2019, categorized by GNRI tertiles, revealed intriguing insights. GNRI exhibited a pronounced gradient across tertiles, with mean values of 97.7 ± 18.8, 115.8 ± 2.7, and 129.5 ± 8.2 in Tertiles 1, 2, and 3, respectively (*p* < 0.001), underscoring its role as a significant factor. Statistically significant differences surfaced among GNRI tertiles in the distribution of age groups, gender, race, marital status, education, and various health parameters. Participants in the lower GNRI tertile demonstrated a proclivity for advanced age, male gender, and a heightened prevalence of comorbidities such as diabetes mellitus, hypertension, and metabolic syndrome. Anthropometric measurements revealed compelling variations, with lower GNRI tertiles associated with diminished height, weight, and BMI. Moreover, the prevalence of coronary heart disease, stroke, chronic obstructive pulmonary disease (COPD), chronic kidney disease (CKD), and mortality displayed marked differences across GNRI tertiles (Table 1).

**Table 1:** Baseline characteristics of participants with osteoporosis and osteopenia in NHANES 2005–2018.

**Characteristics**	**Total (*n* = 6,824)**	**GNRI Tertile1 (*n* = 2,275)**	**GNRI Tertile 2 (*n* = 2,274)**	**GNRI Tertile 3 (*n* = 2,275)**	***P*-value**
GNRI, mean ± SD	114.4 ± 17.7	97.7 ± 18.8	115.8 ± 2.7	129.5 ± 8.2	<0.001
Age, mean ± SD	60.7 ± 15.1	60.5 ± 16.8	61.0 ± 14.8	60.7 ± 13.5	0.653
**Age group**, ***n*** **(%)**					<0.001
<45 years	970 (14.2)	388 (17.1)	317 (13.9)	265 (11.6)	
45–64 years	2,877 (42.2)	838 (36.8)	966 (42.5)	1,073 (47.2)	
≥65 years	2,977 (43.6)	1,049 (46.1)	991 (43.6)	937 (41.2)	
**Gender**, ***n*** **(%)**					<0.001
Female	4,061 (59.5)	1,079 (47.4)	1,274 (56)	1,708 (75.1)	
Male	2,763 (40.5)	1,196 (52.6)	1,000 (44)	567 (24.9)	
**Race**, ***n*** **(%)**					<0.001
Non-Hispanic White	3,649 (53.5)	1,217 (53.5)	1,253 (55.1)	1,179 (51.8)	
Non-Hispanic Black	799 (11.7)	330 (14.5)	225 (9.9)	244 (10.7)	
Mexican American	1,079 (15.8)	252 (11.1)	348 (15.3)	479 (21.1)	
Other Hispanic	583 (8.5)	153 (6.7)	203 (8.9)	227 (10)	
Other race—Including multi-racial	714 (10.5)	323 (14.2)	245 (10.8)	146 (6.4)	
**Marriage**, ***n*** **(%)**					<0.001
Single	603 (8.8)	224 (9.8)	193 (8.5)	186 (8.2)	
Married	3,714 (54.4)	1,198 (52.7)	1,297 (57)	1,219 (53.6)	
Divorced/widowed/separated	2,482 (36.4)	834 (36.7)	779 (34.3)	869 (38.2)	
**Education**, ***n*** **(%)**					<0.001
Less than high school	1,938 (28.4)	654 (28.7)	604 (26.6)	680 (29.9)	
High school grad/GED or equivalent	1,664 (24.4)	558 (24.5)	541 (23.8)	565 (24.8)	
Some college or AA degree	1,745 (25.6)	522 (22.9)	560 (24.6)	663 (29.1)	
College graduate or above	1,465 (21.5)	536 (23.6)	564 (24.8)	365 (16)	
Height (cm), mean ± SD	163.9 ± 9.8	165.7 ± 9.7	164.5 ± 9.8	161.5 ± 9.5	<0.001
Weight (kg), mean ± SD	72.1 ± 16.2	62.2 ± 12.3	70.6 ± 12.6	83.3 ± 15.8	<0.001
BMI (kg/m^2^), mean ± SD	26.8 ± 5.2	22.5 ± 3.4	25.9 ± 2.7	31.8 ± 4.5	<0.001
**Coronary heart disease**, ***n*** **(%)**					<0.001
No	6,229 (91.3)	2,047 (90)	2,083 (91.6)	2,099 (92.3)	
Yes	431 (6.3)	144 (6.3)	148 (6.5)	139 (6.1)	
**DM**, ***n*** **(%)**					<0.001
No	4,802 (70.4)	1,758 (77.3)	1,632 (71.8)	1,412 (62.1)	
IFG	331 (4.9)	85 (3.7)	112 (4.9)	134 (5.9)	
IGT	323 (4.7)	102 (4.5)	101 (4.4)	120 (5.3)	
DM	1,368 (20.0)	330 (14.5)	429 (18.9)	609 (26.8)	
**Hypertension**, ***n*** **(%)**					<0.001
No	3,249 (47.6)	1,250 (54.9)	1,110 (48.8)	889 (39.1)	
Yes	3,574 (52.4)	1,025 (45.1)	1,164 (51.2)	1,385 (60.9)	
**Stroke**, ***n*** **(%)**					<0.001
No	6,275 (92.0)	2,057 (90.4)	2,114 (93)	2,104 (92.5)	
Yes	407 (6.0)	143 (6.3)	126 (5.5)	138 (6.1)	
**COPD**, ***n*** **(%)**					<0.001
No	6,433 (94.3)	2,073 (91.1)	2,168 (95.3)	2,192 (96.4)	
Yes	246 (3.6)	122 (5.4)	73 (3.2)	51 (2.2)	
**CKD**, ***n*** **(%)**					<0.001
No	4,858 (71.2)	1,437 (63.2)	1,710 (75.2)	1,711 (75.2)	
Yes	1,710 (25.1)	600 (26.4)	555 (24.4)	555 (24.4)	
**Parkinson's disease**, ***n*** **(%)**					0.377
No	6,721 (98.5)	2,247 (98.8)	2,242 (98.6)	2,232 (98.1)	
Yes	98 (1.4)	26 (1.1)	31 (1.4)	41 (1.8)	
**Metabolic syndrome**, ***n*** **(%)**					<0.001
No	4,464 (65.4)	1,990 (87.5)	1,516 (66.7)	958 (42.1)	
Yes	2,360 (34.6)	285 (12.5)	758 (33.3)	1,317 (57.9)	
**Mortality**, ***n*** **(%)**					<0.001
Alive	5,468 (80.3)	1,662 (73.2)	1,849 (81.5)	1,957 (86.1)	
Dead	1,342 (19.7)	608 (26.8)	419 (18.5)	315 (13.9)	
**Death cause**, ***n*** **(%)**					<0.001
Cardiovascular disease	341 (5.0)	159 (7)	104 (4.6)	78 (3.4)	
Cancer	312 (4.6)	132 (5.8)	96 (4.2)	84 (3.7)	
Alzheimer's disease	59 (0.9)	32 (1.4)	15 (0.7)	12 (0.5)	
Cerebrovascular diseases	82 (1.2)	31 (1.4)	29 (1.3)	22 (1)	
Chronic lower respiratory diseases	89 (1.3)	47 (2.1)	28 (1.2)	14 (0.6)	
Diabetes mellitus	32 (0.5)	11 (0.5)	10 (0.4)	11 (0.5)	
Influenza and pneumonia	29 (0.4)	13 (0.6)	12 (0.5)	4 (0.2)	
Nephritis, nephrotic syndrome and nephrosis	27 (0.4)	15 (0.7)	7 (0.3)	5 (0.2)	
Accidents (unintentiol injuries)	25 (0.4)	12 (0.5)	8 (0.4)	5 (0.2)	
All other causes	346 (5.1)	156 (6.9)	110 (4.8)	80 (3.5)	
Follow time (years), mean ± SD	7.9 ± 4.3	7.5 ± 4.4	8.2 ± 4.3	8.0 ± 4.2	<0.001

Notably, the impact of GNRI on mortality was investigated over a mean follow-up period of 7.9 ± 4.3 years. Among the 6,824 participants, 1,342 (19.7%) were reported deceased during this interval. Stratification by GNRI tertiles unveiled distinct mortality rates: 26.8% in the lowest tertile, 18.5% in the middle tertile, and the lowest mortality rate of 13.9% in the highest GNRI tertile. Causes of death exhibited variability across tertiles, with CVD disease and cancer emerging as prominent contributors to mortality (Table 1).

### Inverse J-shaped association between GNRI and mortality in patients with osteoporosis and osteopenia

**Figure 1:**
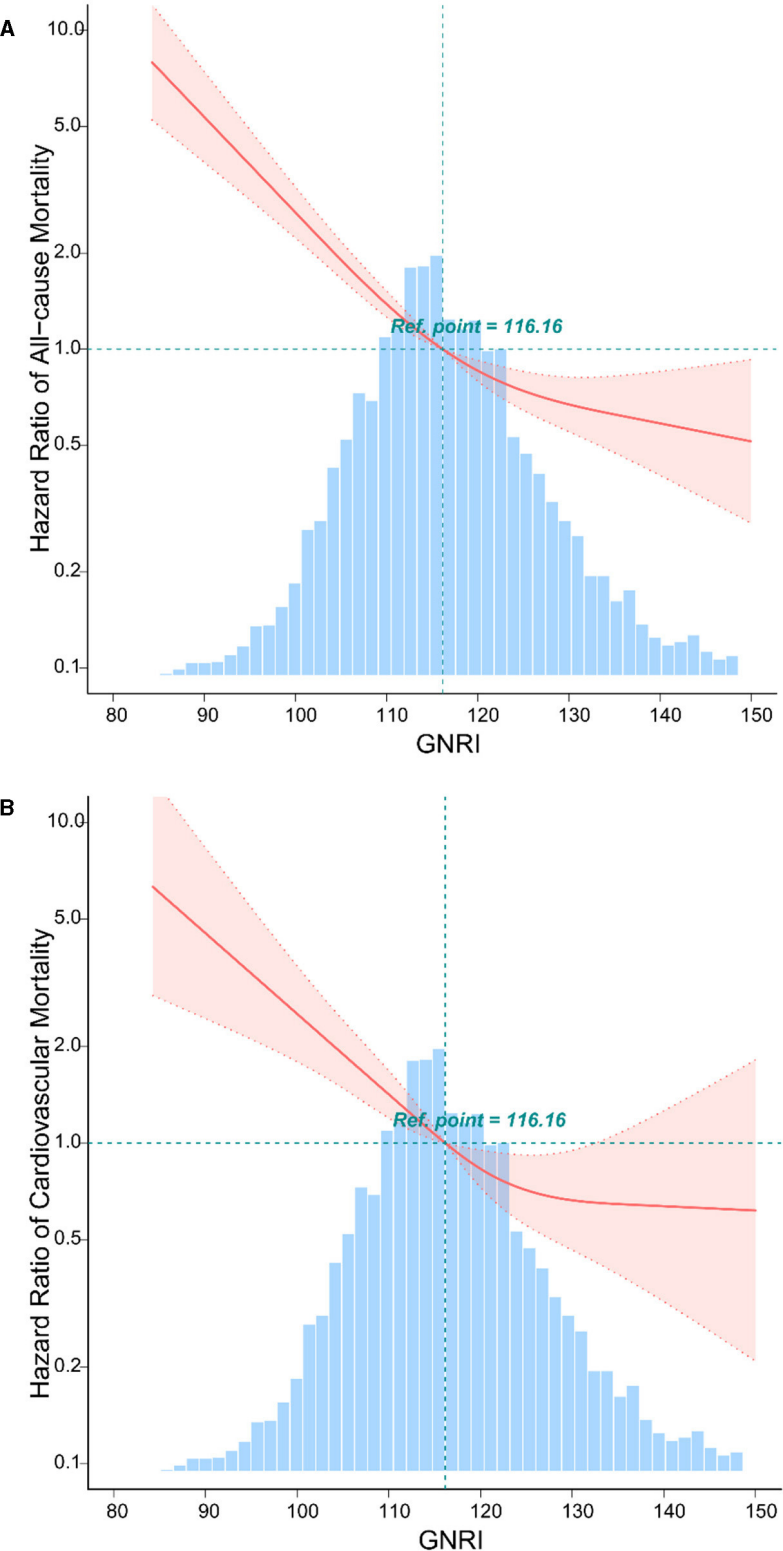
The relationship between GNRI with all-cause, and CVD mortality by curve fitting in osteoporosis and osteopenia. Adjusted for gender, race, age, marital status, body mass index, education, diabetes mellitus, hypertension, coronary heart disease, chronic kidney disease, metabolic syndrome, and stroke. **(A)** The curve fitting of GNRI and all-cause mortality in osteoporosis and osteopenia. **(B)** The curve fitting of GNRI and CVD mortality in osteoporosis and osteopenia.

**Figure 2:**
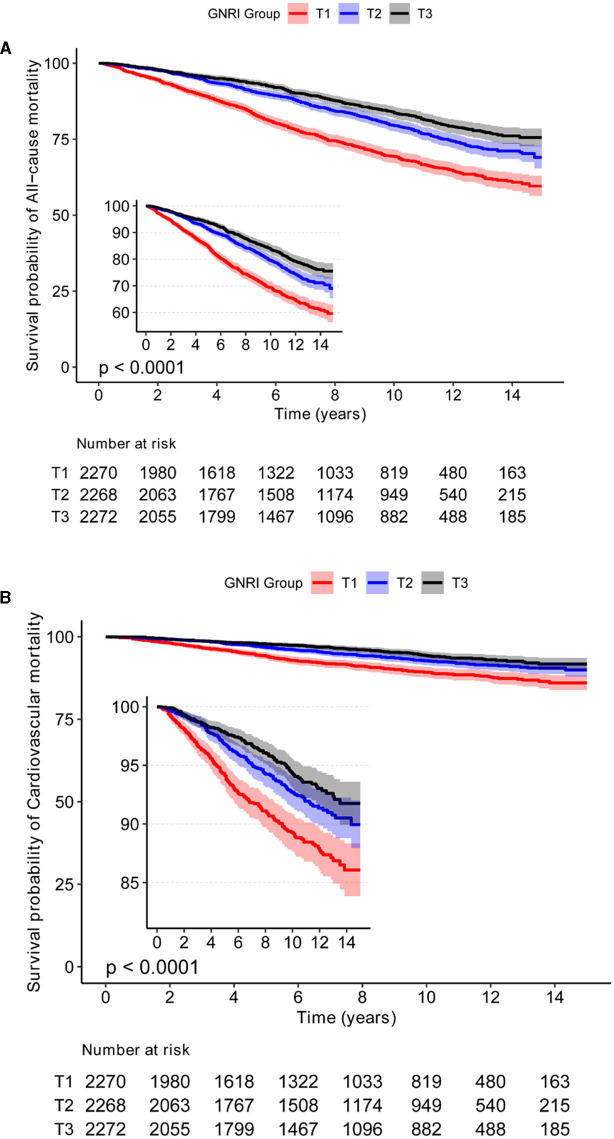
The relationship between GNRI with all-cause, and CVD mortality by Kaplan-Meier survival curves in Osteoporosis and Osteopenia. **(A)** The KM curve of GNRI and all-cause mortality in osteoporosis and osteopenia. **(B)** The KM curve of GNRI and CVD mortality in osteoporosis and osteopenia.

The analysis reveals a distinctive inverse J-shaped relationship between the GNRI and both all-cause and CVD mortality in individuals diagnosed with osteoporosis and osteopenia (Figures 1A, B). Lower GNRI levels are prominently associated with elevated risks of all-cause mortality and CVD events, whereas higher GNRI levels exhibit a notable reduction in mortality risk. Kaplan-Meier survival curves further underscore this intricate association, depicting a compelling correlation between incremental GNRI levels and a statistically significant decrease in both all-cause mortality and CVD risk (*P* < 0.05) (Figures 2A, B). This graphical representation emphasizes the clinical relevance of GNRI in risk stratification for individuals with osteoporosis and osteopenia, offering valuable insights into their mortality outcomes.

#### Hazard ratios for all-cause and CVD mortality in participants with osteoporosis and osteopenia

##### All-cause mortality

In examining all-cause mortality among individuals with osteoporosis and osteopenia, stratified by GNRI tertiles, notable associations emerged. The unadjusted model (Model 1) revealed a significant 2-fold increase in mortality risk for the lowest GNRI tertile compared to the reference group (HR = 2.07, 95% CI: 1.81–2.37, *p* < 0.001). Adjustments for gender, body mass index, and age in Model 2 sustained this association (HR = 1.78, 95% CI: 1.44–2.20, *p* < 0.001), a trend that persisted through subsequent adjustments in Models 3 and 4, encompassing comprehensive demographic and clinical factors (HR = 1.69, 95% CI: 1.37–2.09, *p* < 0.001; HR = 1.60, 95% CI: 1.29–1.99, *p* < 0.001, respectively). The trend test across GNRI tertiles underscored a significant, consistent trend of increasing mortality risk with lower tertiles (Trend test HR = 1.46, 95% CI: 1.36–1.56, *p* < 0.001) (Table 2).

**Table 2:** The HRs of GNRI with all-cause, and CVD mortality in the participants with osteoporosis and osteopenia.

**Variable**	**Total**	**Event (%)**	**Model 1**	***P*-value**	**Model 2**	***P*-value**	**Model 3**	***P*-value**	**Model 4**	***P*-value**
**All-cause mortality**										
GNRI Tertile3	2,275	315 (13.8)	1 (Ref)		1 (Ref)		1 (Ref)		1 (Ref)	
GNRI Tertile2	2,274	419 (18.4)	1.29 (1.12–1.49)	0.001	1.13 (0.95–1.35)	0.173	1.11 (0.93–1.32)	0.242	1.05 (0.88–1.25)	0.582
GNRI Tertile1	2,275	608 (26.7)	2.07 (1.81–2.37)	<0.001	1.78 (1.44–2.2)	<0.001	1.69 (1.37–2.09)	<0.001	1.6 (1.29–1.99)	<0.001
Trend test	6,824	1,342 (19.7)	1.46 (1.36–1.56)	<0.001	1.38 (1.25–1.53)	<0.001	1.34 (1.21–1.49)	<0.001	1.31 (1.17–1.46)	<0.001
**Cardiovascular mortality**										
GNRI Tertile3	2,275	100 (4.4)	1 (Ref)		1 (Ref)		1 (Ref)		1 (Ref)	
GNRI Tertile2	2,274	133 (5.8)	1.3 (1–1.68)	0.049	1.15 (0.85–1.58)	0.365	1.13 (0.82–1.54)	0.457	1.07 (0.78–1.46)	0.672
GNRI Tertile1	2,275	190 (8.4)	2.04 (1.6–2.6)	<0.001	1.81 (1.25–2.63)	0.002	1.71 (1.17–2.48)	0.005	1.74 (1.19–2.54)	0.004
Trend test	6,824	423 (6.2)	1.44 (1.28–1.63)	<0.001	1.39 (1.16–1.67)	<0.001	1.35 (1.12–1.62)	0.002	1.37 (1.13–1.65)	0.001

Model 1: Crude Model.

Model 2: Adjusted for gender, body mass index, and age.

Model 3: Adjusted for gender, race, age, marital status, body mass index, and education.

Model 4: Adjusted for gender, race, age, marital status, body mass index, education, diabetes mellitus, hypertension, coronary heart disease, chronic kidney disease, chronic obstructive pulmonary disease (COPD), metabolic syndrome, stroke, and Parkinson's disease.

##### CVD mortality

A similar pattern emerged in CVD mortality across GNRI tertiles. Model 1, the unadjusted analysis, indicated a substantial increase in CVD mortality for the lowest GNRI tertile (HR = 2.04, 95% CI: 1.6–2.6, *p* < 0.001). This association persisted through subsequent adjustments, including Models 2, 3, and 4, which incorporated gender, body mass index, age, and a comprehensive array of demographic and clinical factors (HR = 1.81, 95% CI: 1.25–2.63, *p* = 0.002; HR = 1.71, 95% CI: 1.17–2.48, *p* = 0.005; HR = 1.74, 95% CI: 1.19–2.54, *p* = 0.004, respectively). The trend analysis for CVD mortality highlighted a consistent and significant pattern of heightened risk associated with lower GNRI tertiles (Trend test HR = 1.44, 95% CI: 1.28–1.63, *p* < 0.001) (Table 2).

These findings underscore a robust association between GNRI levels and both all-cause and CVD mortality, emphasizing the pivotal role of nutritional status in individuals with osteoporosis and osteopenia.

#### Subgroup analyses

In this comprehensive exploration of HRs for all-cause and CVD mortality among individuals with osteoporosis and osteopenia, categorized by GNRI tertiles, distinctive patterns surfaced within specific subgroups (Table 3).

**Table 3:** The HRs of GNRI with all-cause, and CVD mortality in subgroup analyses of osteoporosis and osteopenia.

**Subgroup**	**Total**	**All-cause mortality**				**Cardiovascular mortality**			
		**Death event (%)**	**Adj. HR**	**Adj**. ***P-*****value**	***P*** **for interaction**	**CVD death event (%)**	**Adj.HR**	**Adj**. ***P*****-value**	***P*** **for interaction**
**Osteopenia**					0.032				0.216
GNRI Tertile3	2,016	270 (13.4)	1 (Ref)			88 (4.4)	1 (Ref)		
GNRI Tertile2	1,983	338 (17)	1.14 (0.94–1.38)	0.188		113 (5.7)	1.17 (0.84–1.64)	0.363	
GNRI Tertile1	1,876	445 (23.7)	1.62 (1.28–2.05)	<0.001		146 (7.8)	1.65 (1.09–2.48)	0.017	
Trend test	5,875	1053 (17.9)	1.3 (1.16–1.46)	<0.001		347 (5.9)	1.3 (1.06–1.6)	0.01	
**Osteoporosis**									
GNRI Tertile3	259	45 (17.4)	1 (Ref)			12 (4.6)	1 (Ref)		
GNRI Tertile2	291	81 (27.8)	1.04 (0.67–1.61)	0.855		20 (6.9)	0.87 (0.37–2.07)	0.756	
GNRI Tertile1	399	163 (40.9)	1.91 (1.15–3.16)	0.012		44 (11)	1.78 (0.66–4.74)	0.252	
Trend test	949	289 (30.5)	1.52 (1.19–1.93)	0.001		76 (8)	1.54 (0.96–2.47)	0.073	
**Age** **<** **45 years**					0.207				0.751
GNRI Tertile3	265	7 (2.6)	1 (Ref)			3 (1.1)	1 (Ref)		
GNRI Tertile2	317	7 (2.2)	1.46 (0.38–5.61)	0.58		2 (0.6)	2.45 (0.23–26.24)	0.458	
GNRI Tertile1	388	9 (2.3)	1.89 (0.4–8.93)	0.423		3 (0.8)	6.18 (0.56–68.47)	0.137	
Trend test	970	23 (2.4)	1.36 (0.64–2.89)	0.425		8 (0.8)	2.49 (0.78–8.01)	0.125	
**Age 45–64 years**									
GNRI Tertile3	1,073	68 (6.3)	1 (Ref)			13 (1.2)	1 (Ref)		
GNRI Tertile2	966	72 (7.5)	1.16 (0.78–1.74)	0.467		17 (1.8)	1.95 (0.8–4.75)	0.142	
GNRI Tertile1	838	120 (14.3)	2.4 (1.5–3.83)	<0.001		26 (3.1)	3.94 (1.4–11.09)	0.009	
Trend test	2,877	260 (9)	1.64 (1.3–2.07)	<0.001		56 (1.9)	1.99 (1.21–3.29)	0.007	
**Age** ≥**65 years**									
GNRI Tertile3	937	240 (25.6)	1 (Ref)			84 (9)	1 (Ref)		
GNRI Tertile2	991	340 (34.3)	1.08 (0.89–1.32)	0.43		114 (11.5)	0.99 (0.71–1.38)	0.943	
GNRI Tertile1	1,049	479 (45.7)	1.54 (1.21–1.97)	<0.001		161 (15.3)	1.42 (0.94–2.15)	0.092	
Trend test	2,977	1,059 (35.6)	1.28 (1.13–1.44)	<0.001		359 (12.1)	1.24 (1.01–1.52)	0.042	
**Female**					0.073				0.306
GNRI Tertile3	1,708	224 (13.1)	1 (Ref)			71 (4.2)	1 (Ref)		
GNRI Tertile2	1,274	216 (17)	1.21 (0.96–1.53)	0.103		57 (4.5)	1.07 (0.69–1.64)	0.765	
GNRI Tertile1	1,079	248 (23)	2.01 (1.54–2.63)	<0.001		73 (6.8)	1.97 (1.22–3.19)	0.006	
Trend test	4,061	688 (16.9)	1.45 (1.27–1.66)	<0.001		201 (4.9)	1.46 (1.14–1.86)	0.002	
**Male**									
GNRI Tertile3	567	91 (16)	1 (Ref)			29 (5.1)	1 (Ref)		
GNRI Tertile2	1,000	203 (20.3)	0.91 (0.69–1.2)	0.493		76 (7.6)	1.06 (0.66–1.72)	0.801	
GNRI Tertile1	1,196	360 (30.1)	1.26 (0.9–1.76)	0.182		117 (9.8)	1.39 (0.77–2.51)	0.272	
Trend test	2,763	654 (23.7)	1.19 (1–1.4)	0.046		222 (8)	1.21 (0.91–1.62)	0.185	

Adjusted for gender, race, age, marital status, body mass index, and education.

##### Osteopenia and osteoporosis subgroup

Participants in GNRI Tertile 1 showcased a notable surge in both all-cause and CVD mortality with osteopenia, revealing adjusted HRs of 1.62 (95% CI: 1.28–2.05, *p* < 0.001) and 1.65 (95% CI: 1.09–2.48, *p* = 0.017), respectively. Within the osteoporosis subgroup, individuals in GNRI Tertile 1 presented a significant elevation in all-cause mortality risk (HR = 1.91, 95% CI: 1.15–3.16, *p* = 0.012). However, the association with CVD mortality did not reach statistical significance after adjustments (Table 3).

##### Age subgroups

Age <45 years: although trends hinted at heightened mortality risk with lower GNRI tertiles, statistical significance eluded both all-cause and CVD mortality in this age category.

Age 45–64 years: participants in GNRI Tertile 1 displayed a substantial increase in both all-cause and CVD mortality, revealing adjusted HRs of 2.4 (95% CI: 1.5–3.83, *p* < 0.001) and 3.94 (95% CI: 1.4–11.09, *p* = 0.009), respectively (Table 3).

Age ≥ 65 years: within this older age group, individuals in GNRI Tertile 1 encountered a significant elevation in both all-cause and CVD mortality risk (HR = 1.54, 95% CI: 1.21–1.97, *p* < 0.001; HR = 1.42, 95% CI: 0.94–2.15, *p* = 0.092, respectively). The trend test confirmed a consistent escalation in risk with lower GNRI tertiles (Table 3).

##### Gender subgroups

Females: in alignment with overall trends, females in GNRI Tertile 1 bore significantly elevated risks of all-cause and CVD mortality (HR = 2.01, 95% CI: 1.54–2.63, *p* < 0.001; HR = 1.97, 95% CI: 1.22–3.19, *p* = 0.006, respectively) (Table 3).

Males: the trend test indicated a modest increase in all-cause mortality risk with lower GNRI tertiles (HR = 1.19, 95% CI: 1–1.4, *p* = 0.046), but individual associations for CVD mortality were not consistently significant across all models (Table 3).

In addition to the observed interaction between osteopenia and GNRI tertiles in relation to all-cause mortality (*P* = 0.032), no significant interactions were found in other subgroups, indicating that the association between GNRI and mortality outcomes remains consistent across various demographic and clinical categories.

## Discussion

Prior research has consistently supported the correlation between the GNRI and osteoporosis (4, 6). Wang et al.'s study on American postmenopausal women revealed a beneficial link between GNRI and femur bone mineral density, coupled with a negative link to osteoporosis risk (4). Similarly, Huang et al.'s study in the older adult identified GNRI serves as a distinct and significant factor to osteoporosis risk in prior research, illustrating a non-linear negative correlation (6). Our study aimed to elucidate the relationship between GNRI and all-cause mortality as well as CVD mortality in individuals diagnosed with osteoporosis and osteopenia.

The analysis over a mean follow-up of 7.9 years demonstrated a distinctive inverse J-shaped association between GNRI levels and both all-cause and CVD mortality among individuals with osteoporosis and osteopenia, aligning with prior researches in other population (6). Huo et al.'s study, encompassing 10,037 older adult hypertensive patients, showcased the considerable predictive prowess of GNRI for both all-cause and CVD mortality (15). In a distinct cohort, Chai et al. focused on 579 adults with COPD and demonstrated that lower GNRI independently correlated with elevated all-cause mortality risk (16). Additionally, Shen et al.'s research, encompassing 4,400 older Americans with diabetes, r strengthened the reliability of GNRI as a predictor for mortality outcomes (5).Collectively, these studies (5, 15, 16), including our own, underscore the versatility of GNRI as a prognostic marker for mortality, with specific details and robust findings in various health conditions. Importantly, our study contributes specific insights into the unique context of osteoporosis and osteopenia.

Our analysis of HRs for all-cause and CVD mortality underscores the significant impact of the GNRI on survival outcomes. Stratification by GNRI tertiles reveals a consistent dose-response relationship, emphasizing increasing mortality risk with lower tertiles, and these associations remain robust after thorough adjustments for demographic and clinical factors. Importantly, individuals with osteopenia and osteoporosis in the lowest GNRI tertile face a substantial elevation in both all-cause and CVD mortality, highlighting the clinical relevance of GNRI in effectively stratifying risk. Age-specific variations in HRs underscore the importance of tailored nutritional interventions for different age cohorts. Similarly, gender-specific patterns emphasize the need for sex-specific considerations in managing nutritional risk among individuals with compromised bone health. In summary, our findings provide nuanced insights into the differential impact of GNRI on mortality across subgroups, reinforcing its clinical utility in risk stratification and targeted interventions for individuals with compromised bone health.

The GNRI-mortality relationship in individuals with osteoporosis and osteopenia may involve immune function, inflammatory pathways, and overall health status, warranting further mechanistic exploration (17–21). It may be explained by compromised immune function and heightened inflammation due to malnutrition (17–23). This inflammatory state potentially contributes to CVD events, increasing overall mortality risk (24, 25). Additionally, suboptimal nutrition may worsen osteoporosis progression, raising the likelihood of fractures and related complications (24, 25). Age-specific variations underscore the need for tailored nutritional interventions.

Despite the strengths of our study, including a large and diverse cohort, certain limitations merit consideration. The observational nature of our study design precludes establishing causation, and residual confounding factors may influence the observed associations. Future research endeavors should delve deeper into the mechanistic underpinnings of the GNRI-mortality relationship, considering factors such as inflammation, hormonal status, and functional capacity.

In conclusion, our findings underscore the pivotal role of GNRI as a prognostic marker for mortality in individuals with osteoporosis and osteopenia. The nuanced relationship, dose-response pattern, and consistency across subgroups highlight the potential utility of GNRI in clinical practice for risk stratification and targeted interventions to improve outcomes in this vulnerable population.

## Data Availability

Publicly available datasets were analyzed in this study. This data can be found at: https://www.cdc.gov/nchs/nhanes/index.htm.
